# A Tool for Rating the Value of Health Education Mobile Apps to Enhance Student Learning (MARuL): Development and Usability Study

**DOI:** 10.2196/18015

**Published:** 2020-07-31

**Authors:** Tehmina Gladman, Grace Tylee, Steve Gallagher, Jonathan Mair, Sarah C Rennie, Rebecca Grainger

**Affiliations:** 1 Education Unit University of Otago Wellington Wellington New Zealand; 2 University of Otago Wellington Wellington New Zealand; 3 Education Unit Dunedin School of Medicine University of Otago Dunedin New Zealand; 4 Hutt Valley District Health Board Lower Hutt New Zealand

**Keywords:** questionnaire design, medical education, health occupations students, just-in-time learning, self-directed learning, mobile phone, rubric, mobile learning, mobile apps, mhealth, digital learning

## Abstract

**Background:**

To realize the potential for mobile learning in clinical skills acquisition, medical students and their teachers should be able to evaluate the value of an app to support student learning of clinical skills. To our knowledge, there is currently no rubric for evaluation of quality or value that is specific for apps to support medical student learning. Such a rubric might assist students to be more confident in using apps to support their learning.

**Objective:**

The objective of this study was to develop an instrument that can be used by health professional educators to rate the value of a mobile app to support health professional student learning.

**Methods:**

Using the literature, we developed a list of potential criteria for the evaluation of educational app value, which were then refined with a student group using a modified nominal group technique. The refined list was organized into themes, and the initial rubric, Mobile App Rubric for Learning (MARuL, version 1), was developed. iOS and Android app stores were searched for clinical skills apps that met our inclusion criteria. After the 2 reviewers were trained and the item descriptions were refined (version 2), a random sample of 10 included apps, 5 for each mobile operating system, was reviewed. Interitem and interrater analyses and discussions with the reviewers resulted in refinement of MARuL to version 3. The reviewers completed a review of 41 clinical skills mobile apps, and a second round of interitem and interrater reliability testing was performed, leading to version 4 of the MARuL.

**Results:**

Students identified 28 items (from an initial set of 144 possible items) during the nominal group phase, and these were then grouped into 4 themes: teaching and learning, user centered, professional, and usability. Testing and refinement with reviewers reduced the list to 26 items. Internal consistency for MARuL was excellent (α=.96), and the interrater reliability as measured by the intraclass correlation coefficient (ICC) was good (ICC=0.66).

**Conclusions:**

MARuL offers a fast and user-friendly method for teachers to select valuable apps to enhance student learning.

## Introduction

### Background

Smartphones and tablets have made mobile learning an important component of education, enabling learning anywhere, at any time, using mobile apps [1]. Most education apps have specific functions or aims, such as providing resources for reference while learning or in practice, learning activities and games, or organizing activities related to learning. Apps can be found by keyword search in the app stores (eg, Google Play Store, iOS App Store), through recommendations, or within app store–determined categories [2].

In medical education, the use of apps for reference and learning on the go (*just in time*) for ongoing professional development is widespread [1,3]. However, previous work suggests that medical students may still prefer textbooks and lectures as a learning resource [4,5] and predominantly use apps for reference and revision [1,3,4,6]. Although the determinants of preference for textbooks have not been explored, there may be barriers to finding apps that are both relevant and valuable for student learning [5]. Within app stores, star ratings and reviews are the main indicators of app quality or value; however, these can be subjective and only relevant if the reviewer has needs and expectations similar to the potential app user [7,8]. Furthermore, a search of medical apps reveals a very large number of potential apps to choose from and many that have not received enough reviews to get a rating. Owing to the number of options to choose from, and the lack of good quality guidance to inform their choices, students may not be accessing and using apps that could support their learning. Providing a standard way of evaluating apps for medical education may improve students’ ability to find or identify apps to support their learning.

### Evaluating Apps

To avoid the app overload caused by the increasing number of apps in the app stores, potential app users are recommended to use the literature to identify valuable apps [2,9,10]. There is evolving literature aiming to identify apps for health conditions; to support self-management, education, or behavior change [11,12]; and to evaluate app quality [10,13]. To date, similar literature in education has focused on apps for use by teachers working with students with learning disabilities [14] or preschool student learning [15]. In addition to providing curated lists of apps, this literature often evaluates app quality with instruments that include some generic aspects of proposed quality; for example, a component to evaluate the suitability of design and aesthetics. However, these rubrics also have components or domains specific to the type or main function of an app. For instance, the evaluation rubric for health care smartphone apps by Jin and Kim [11] emphasizes the input of medical experts and the developers’ citing of authoritative sources. In comparison, the rubric by Lee and Kim [12] for evaluating educational apps emphasizes the *teaching and learning* component of an app. When developing a rubric to evaluate apps, a balance between general and specific criteria is needed to ensure that the rubric is both reliable and objective [10]. To our knowledge, there is currently no rubric for evaluation of quality or value that is specific for apps to support medical student learning. Such a rubric might assist students to be more confident in using apps to support their learning.

### App Evaluation Rubrics

Most multidimensional app evaluation rubrics include both user-centered features (eg, engagement) and technology-centered features (eg, functionality) [10]. Although the model for education app evaluation by Lee and Kim [12] emphasizes teaching and learning, it also recognized the need for a technologically stable app, with “quick interactions (fast loading times) and error-free stability” [12]. It was concluded that factors must be considered together because they are interdependent in nature rather than independent. Stoyanov et al [10] also emphasized the importance of considering multiple features of an app to increase the objectivity and reliability of an app evaluation scale.

An additional factor that is relevant for health-related mobile apps is the credibility of the app content. Having apps with evidence-based approaches to health should minimize the risk of harm and promote safe application of knowledge by app users [16-18]. In the medical education setting, for example, when learning clinical skills, it is critical to learn skills that are relevant and accurate in the local health care environment. Apps that focus on clinical skills—that is, apps supporting learning of history taking, examination, communication, or procedures—provide opportunities for just-in-time learning in clinical settings where textbooks are not available, making apps supporting acquisition of clinical skills a good test case for the development of an app evaluation rubric.

### Purpose and Research Question

To realize the potential for mobile learning in clinical skills acquisition, medical students and their teachers should be able to evaluate the value of an app for supporting the learning of clinical skills. Our aim was to develop a rubric that can be used by staff to evaluate the value of apps for learning the clinical skills of history taking, examination, communication, or procedures so that recommendations can be made to students. The initial research question was as follows: “What key domains of value need to be included in a reliable measure that teachers can use to rate mobile apps for just-in-time learning?”

## Methods

### Overview

The development of evaluative rubrics for apps uses some or all of the following steps. A literature search is completed to identify previous literature with quality evaluation criteria, followed by a discussion among topic experts to group criteria and develop scale items [10]. A survey or focus group is conducted to gain expert opinion on the relevance of items generated from the literature search [12]. Users and experts evaluate apps with the draft rubric to test its reliability and validity [11,12], with items in the rubric refined accordingly. Many rubrics are structured using overarching factors with relevant subterms to clarify and give examples [10,11,19].

**Figure 1:**
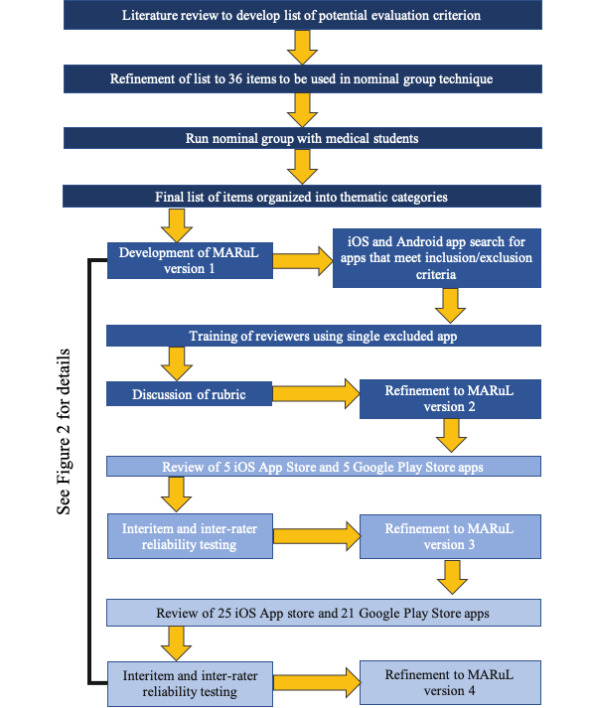
Process for developing the Mobile App Rubric for Learning. MARuL: Mobile App Rubric for Learning.

We undertook a literature review to identify potential rubric items, which were evaluated by a group of medical students using a modified nominal group technique. The performance of the preliminary rubric was then evaluated by clinical and education experts for use with clinical skills apps, which were identified using a comprehensive search strategy in Google Play and iTunes app stores. The rubric was then refined through expert feedback and statistical analysis using the classical test theory (Figure 1).

### Definitions

We defined *value* as referring to an object that is perceived to have utility in meeting short- or long-term goals [20]. *Just-in-time learning* is defined as a method of learning that is driven by the learner, when and where they require it [21]. With this definition, the learner anticipates learning and performance requirements rather than responding to them [21]. Just-in-time learning occurs temporally close to a clinical learning encounter, for example, with a mobile device [22,23]. *Clinical skills* apps are those that include opportunities to learn and improve history taking, examination, communication, or procedures [24]. A *rubric* is a scoring method that uses evaluative criteria with quality definitions and a clear scoring strategy to determine the quality of the global concept being scored [25].

### Development of Potential Rubric Items

A literature review was undertaken to identify criteria previously used to evaluate the quality of mobile apps for education. An expert librarian conducted a literature search on Scopus, MEDLINE, and Google Scholar (between August 14 and 21, 2018) using search terms related to the evaluation of educational mobile apps using evidence-based methods (Textboxes 1-4). After duplicates were removed, the records were exported into Rayyan QCRI (Qatar Computing Research Institute), a cloud-based method for completing systematic reviews of the literature [26], and 4 educational experts (TG, SR, SG, and RG) independently reviewed the titles and abstracts of the records to identify articles that potentially met the inclusion criteria. The inclusion criteria used were as follows: clear descriptions of the concepts, list, or rubric for the evaluation of app quality; reliability or validity testing of the method of evaluation; and the evaluation was for educational mobile apps, which we defined as apps whose primary purpose was to support the education of any population. Articles were excluded if they were literature reviews or they described a framework for evaluation without describing a specific measure. Articles were included if 3 or more reviewers agreed on their relevance.

**Device related literature search terms.**

- app
- smart*
- phone*
- cellular
- smartphone
- smart phone
- mobile
- tablet

**Action related literature search terms.**

- evaluat*
- assess*
- apprais*
- measure*
- validat*
- test*
- determine*
- measur*

**Measure related literature search terms.**

- criteria
- checklist*
- rubric*
- framework*
- quality
- useful*

**Teaching and learning related literature search terms.**

- educat*
- learn*
- teach*
- student*
- education*
- learning*
- educate

The full texts of all identified relevant articles were reviewed in detail by a reviewer (GT), and the concepts or terms that measure or assess app quality were extracted along with definitions. The extracted terms and definitions were then grouped and organized by GT, a medical student representing the end user for the rubric, and TG, an academic with expertise in electronic learning (e-learning) and educational psychology. Overarching concepts (called *categories*) were identified and related terms were grouped together within the concepts. For instance, *use without an internet connection* was grouped with *platform*, *syncing*, *updates*, and *compatibility* under the concept of *technical specifications*. Similar terms were merged.

### Nominal Group Technique

A convenience sample of 10 medical students, recruited using targeted invitations, participated in a nominal group held at the University of Otago Wellington, School of Medicine, on December 6, 2018. Ethical approval was obtained from the Otago Human Ethics Committee, reference number D18/337, and written consent was obtained from the students.

In the nominal group technique, group interaction is facilitated by the leader and verbal interaction is restricted to a discussion between the leader and participants with no discussion between participants [27]. The technique is particularly useful in ensuring that all members of a group are heard. This makes it an ideal method in a group where there are varied levels of experience with the topic under discussion. Ranking of ideas occurs using votes or a Likert-type scale [27]. A voting system with a predetermined maximum of 3 rounds of voting was used after the initial ranking of terms. Limited voting was chosen to reduce the possibility of participant fatigue [28].

Students were asked to review the refined list of terms/concepts for app evaluation (with definitions) and indicate their top 20 terms on a ranking sheet without discussion. They were also asked to add any important missing terms or concepts. Each student then in turn stated a term on their list not previously offered by a student, which was recorded by the leader. This continued until all terms from each student’s top 20 had been recorded.

Students were then given an opportunity for discussion before the first round of voting. Students voted on item inclusion in the final list using the options *keep*, *unsure*, and *discard*. Overall, 7 or more votes for the same option were considered a majority. Items that did not receive a majority vote were recorded and discussed before the next voting round. In the second round, students were encouraged to vote *keep* or *discard* but still had the option of *unsure*. In the second and third rounds, having 6 or more votes was considered a majority. In the third round, only options *keep* and *discard* were allowed. Results from each round were recorded by a group facilitator, whereas the leader facilitated group discussions and answered questions.

### Development of Rubric

The terms chosen by the nominal group made up the preliminary rubric, which we named *Mobile App Rubric for Learning* (MARuL). Two authors (TG and GT) grouped the terms into themes separately, and then came together to discuss final category names and grouping of terms, with subterms used to develop descriptors for the scale.

### App Search

The search of the iOS and Google Play stores for clinical skills apps was undertaken from January 15 to February 1, 2019. The search was conducted based on the Preferred Reporting Items for Systematic Reviews and Meta-analyses guidelines for systematic reviews, with search terms confirmed by discussion (GT, TG, and RG) after preliminary searches (Textbox 5) [29,30]. The inclusion criteria were as follows: available in English; includes at least one of the keywords in the title or description; includes an interactive element requiring some form of user input; target audience includes medical students; and support for iOS 10 or later/Android version 7 or later. The exclusion criteria were as follows: priced over NZ $10.00 (US $6.30) for a one-off price or monthly subscription (based on expected student willingness to pay); reference-only apps (passive with no student input); designed for staff-only use in formative or summative assessment contexts; were a complement to other software (not standalone; requires a log-in/sign up).

**App search terms.**

- clinical skills
- OSCE
- objective structured clinical examination
- medical history taking
- clinical history taking
- patient history
- medical examination
- physical examination
- clinical examination
- clinical exam
- physical exam
- medical exam
- planning and explaining
- patient education

A data screening and extraction spreadsheet was developed and refined by 2 authors (GT and TG) using Airtable [31]. The app search of the iOS and Google Play Store was conducted independently by 2 authors (GT and TG). Apps were screened based on their title and the description in the app store. App name, developer, operating system, reviewer, and inclusion decision were recorded for all screened apps. For the excluded apps, the first identified exclusion criterion was recorded. GT and TG then reviewed all the apps with discrepant decisions and reached a consensus through discussion.

### Testing and Refinement of the Rubric

To initially test the reliability of the MARuL, we developed a protocol based on previous research [10,30] and trialed this with a randomly chosen subset of 10 apps included from the search. The 10 randomly selected apps, 5 Android apps and 5 iOS apps, were downloaded between May 20 and 22, 2019. The iPhones used were iPhone 6 and 6 plus running iOS 12.3.1. The Android phones were Samsung Galaxy J1Ace running Android 5.1.1. Following the initial testing, we refined the rubric and completed reliability testing with the remaining included apps identified in our search of the app stores. Trialing of the rubric with clinical skills apps was completed independently by 2 authors: (1) a hospital-based junior doctor (3 years postgraduate) training in internal medicine (JM) and (2) the e-learning facilitator for the clinical years at a different campus of our medical school (SG). The first reviewer was able to review the apps from the point of view of a near-peer teacher of medical students, whereas the second reviewer reviewed from the point of view of a learning expert with over 10 years of experience in medical education.

For training, the reviewers first used the MARuL to evaluate a previously excluded app, Clinical Skills by George Sim on iOS. The app was excluded because of a lack of interactive elements. The 2 reviewers each downloaded the app on an iPhone running iOS 12.2, tested the app features for 10 min and then independently evaluated the app using the MARuL. Following their review, they met with TG via videoconferencing to discuss their scoring of the app and ensure their understanding of the rubric items and process.

Once the reviewers (JM and SG) had a clear understanding of the items and process for reviewing the apps, they each downloaded the same 5 randomly selected apps for both mobile operating systems. They independently spent a minimum of 10 min using each app before evaluating the app with the MARuL. MARuL ratings for each app and time taken to complete MARuL rating were collected using Qualtrics (Qualtrics, Provo, UT) [32,], downloaded to an Excel spreadsheet, and analyzed using RStudio and appropriate packages [33-36].

Internal consistency and interrater reliability were calculated, and then a discussion was held via videoconferencing to review discrepancies in scoring on the rubric and identify any refinements required to the items of the MARuL. The MARuL was then revised to rewrite some of the descriptors and remove an item that was considered redundant by the reviewers. The remaining apps were then independently trialed and evaluated using the revised MARuL.

We calculated the internal consistency of the categories and the overall value measure using Cronbach alpha. Cronbach alpha measures how interrelated a set of items in a scale are, with scores ranging from 0 to 1, and higher scores indicating a stronger interitem relationship [37]. Interrater reliability of the categories and overall value measure was calculated using the intraclass correlation coefficient (ICC). The ICC measures how much of the difference between sets of scores is because of measurement error, and it ranges from 0 to 1, with higher scores indicating stronger interrater reliability [38].

## Results

### Rubric Items

The literature search yielded 193 unique articles. After reviewing the titles and abstracts, 134 articles were eliminated. Furthermore, 8 articles were removed following full-text review. From the remaining 51 articles, 144 quality criteria were extracted, including main and descriptive subterms, 69 of which were main terms. After the consensus discussion and deletion of overlapping items and organization, a list of 36 main terms from 46 articles remained. (Multimedia Appendix 1 has the full list of terms and references).

### Nominal Group

The nominal group had 10 students (6 female and 4 male) who had completed 2 years (n=2), 3 years (n=1), or 4 years (n=7) of medical school. Among them, 6 students identified as New Zealand European/Pākehā, 3 as Māori, and 1 as Sri Lankan. The nominal group ranking produced a list of 35 of the 36 terms; the only term excluded from voting was “product description.” Following the first vote, 18 items were kept and 17 were to be voted on again. The second vote on the 17 terms resulted in 10 being kept, 3 discarded, and 4 to be voted on again. After the third vote, 1 term was discarded and 3 received an equal number of keep and discard votes. These terms were “sharing,” “motivation,” and “self-directedness.” The final list of terms voted on by medical students included 28 items. Table 1 shows the initial set of items and the outcome of the initial ranking and 3 voting rounds.

**Table 1:** Outcome of nominal group votes.

Term	Initial ranking	First round of vote	Second round of voting	Third round of voting
Satisfaction	Yes	Keep	—^a^	—
Ease of use	Yes	Keep	—	—
Perceived usefulness	Yes	Keep	—	—
Information quality	Yes	Keep	—	—
Functionality	Yes	Keep	—	—
Engagement	Yes	Keep	—	—
In line with professional standards	Yes	Keep	—	—
Relevance to course	Yes	Keep	—	—
Credibility of developers	Yes	Keep	—	—
Privacy of information	Yes	No decision	Discard	—
Cost	Yes	Keep	—	—
Advantage of using app	Yes	Keep	—	—
Efficiency	Yes	Keep	—	—
Instructional features	Yes	No decision	Keep	—
Capacity to generate learning	Yes	Keep	—	—
Aesthetics	Yes	No decision	Keep	—
Quantity of information	Yes	No decision	Keep	—
User ratings	Yes	No decision	Discard	—
Intention to reuse	Yes	Keep	—	—
Technical specifications	Yes	Keep	—	—
Feedback	Yes	No decision	Keep	—
Pedagogy	Yes	Keep	—	—
Perceived enjoyment	Yes	No decision	No decision	Discard
Perceived importance	Yes	No decision	Keep	—
Subjective quality	Yes	No decision	Keep	—
Sharing	Yes	No decision	No decision	No decision-Discard
Motivation	Yes	No decision	No decision	No decision-Discard
Transparent	Yes	No decision	Keep	—
User experience	Yes	Keep	—	—
Purpose	Yes	No decision	Keep	—
Self-directedness	Yes	No decision	No decision	No decision-Discard
Playfulness	Yes	No decision	Discard	—
Lack of ads	Yes	Keep	—	—
Differentiation	Yes	No decision	Keep	—
User interactivity	Yes	No decision	Keep	—
Product description	No	—	—	—

^a^The decision taken at each round of voting is shown. The voting round where a Keep or Discard decision is made ends the decision making for that item.

### Development of Rubric

The 28 items on the list determined by the student nominal group were grouped by 2 authors (GT and TG) into 4 themes based on their similar definitions and the aspects of value they appeared to measure. The categories formed were user-centered measures (n=7), teaching and learning measures (n=9), professional measures (n=4), and usability measures (n=8).

Each category consists of a set of items that are described by posing questions for the user to consider. The questions were developed using definitions for the terms (written by GT) and the authors’ perspective on what was most important with regard to that measure. After consulting the literature, a 5-point Likert-type scale was chosen as the rating tool (0=does not fulfill the item requirements, 1=poorly fulfills requirements, 2=somewhat fulfills requirements, 3=mostly fulfills requirements, and 4=fully meets requirements) with the descriptors for each point on the scale written to answer the item questions. The rubric scale descriptors were developed by 2 authors (TG and GT) with reference to the literature. Some items were adapted from other rating scales such as the Mobile App Rating Scale (MARS) [10], and these are clearly acknowledged on the final rubric (Multimedia Appendix 2). Scores for each item on the initial rubric are added to give a total score out of 112. Scores are used to classify apps as not at all valuable (<50), potentially valuable (51-69), and probably valuable (>69).

### App Search

A total of 1291 iOS apps and 4190 Android apps were screened by title and description in the Apple and Google Play Stores, respectively. In iOS, 81 apps from 14 search terms (Textbox 5) were identified before removal of duplicates. After removal of duplicates, 35 unique apps were included for evaluation. For Android apps, the search terms identified 106 apps, of which 29 were unique. Apps found by only 1 researcher were not included because of concerns about the consistency of access to the app using typical search criteria. This gave a total of 64 apps with which to test the MARuL rubric.

### Reliability Analysis

Initial testing and review of the rubric using the 10 selected apps occurred between June 18 and 22, 2019. One of the Android apps was unable to be tested by one of the reviewers as it did not run after installation on the device provided for testing and was excluded from further analysis.

**Figure 2:**
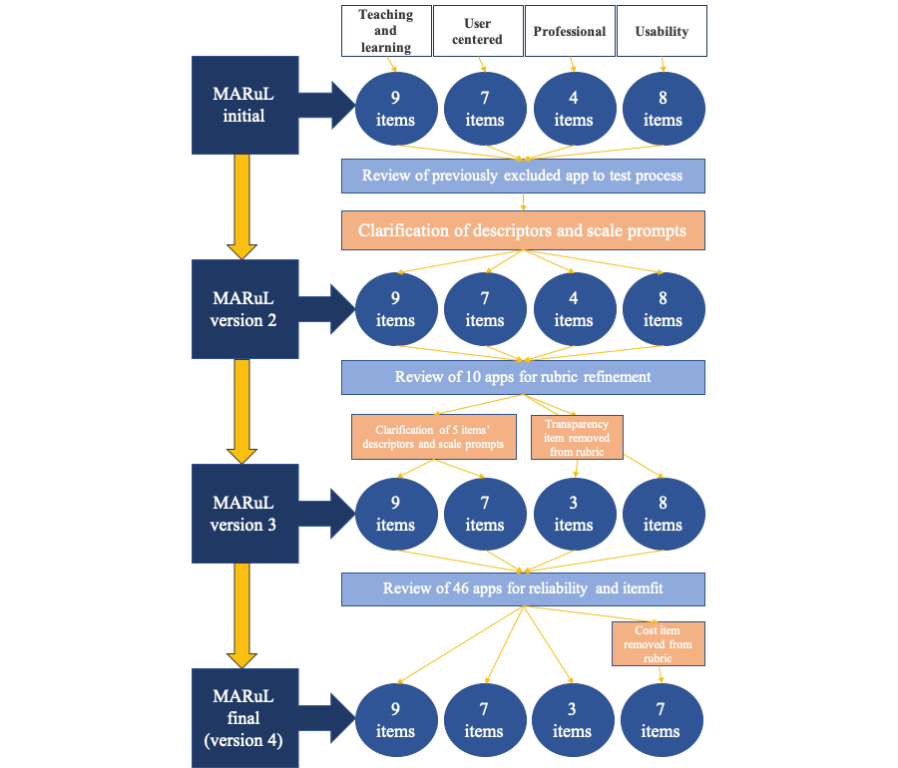
Rubric development process. MARuL: Mobile App Rubric for Learning.

Cronbach alpha for the overall value was excellent (α=.95), and for each of the categories was acceptable to excellent (α=.78-.96). The ICC for overall value was good (ICC=0.81) and moderate to good for each of the categories (ICC=0.71-0.85). Pearson correlations showed moderate-to-strong correlations between the categories (r=0.49-0.91; Table 2). Through analysis and discussion with the reviewers, a further refinement of the descriptors of 5 of the items was completed and 1 item (transparency) was removed from the professional category. In addition, 2 further items (cost and advertisements) in the usability category were considered for removal because of poor statistical performance, but the reviewers felt they should remain as they were likely to be of more immediate concern both to students and to those individuals considering the value of apps for learning. Figure 2 shows the process of development of the MARuL rubric from the initial rubric to the final version.

Version 3 of the MARuL rubric, tested with the remaining 54 apps, consisted of 27 items across 4 categories: user-centered measures (n=7), teaching and learning measures (n=9), professional measures (n=3), and usability measures (n=8). The 54 remaining apps were downloaded and tested between July 29 and October 8, 2019. Of the 54 apps for which testing was attempted, only 46 (25 iOS and 21 Android) could be tested completely for review. As the search took place, 7 apps were removed from their respective app stores and 1 app had updated to require sign up, one of the exclusion criteria, to be used. However, as 41 apps was the minimum sample size needed to determine interrater reliability with 90% assurance, analysis was continued [37].

The mean time to complete the MARuL rubric for each app was 8 min (SD 0.69). The 2 reviewers showed completion times ranging from 1 to 25 min (JM) and 1 to 33 min (SG). The median review time for each reviewer was 6.6 min (JM) and 4.1 min (SG). The discussion with the 2 reviewers indicated that in a small number of cases, there were challenges in evaluating the app, including difficulties with installation or using the app, which accounted for the longer evaluation times.

Cronbach alpha for the overall value was excellent at .96. The categories showed good-to-excellent internal consistency (α=.70-.96). Pearson correlations between the categories were moderate to strong (r range 0.58-0.90). Interrater reliability was also fair to good, with an overall value ICC (2-way) of 0.66 and categories with ICC ranging from 0.45 to 0.75.

From the internal consistency results, it was determined that the item for *cost* was performing in the reverse of its expected direction and did not add useful information to the usability category. It was removed from the rubric, leaving the usability category with 7 items, and the analyses were rerun. With cost removed, the alpha for the usability category improved from .70 to .82 (see Table 3 for full details of all categories).

**Table 2:** Reliability statistics for the initial version of the Mobile App Rubric for Learning after review of 10 apps.

Rubric categories	Intraclass correlation coefficient score^a^	Cronbach α^b^	Pearson r^c^			
			Teaching and learning	User centered	Professional	Usability
Teaching and learning	0.85	.89	1.00	0.91	0.83	0.72
User centered	0.78	.96	N/A^d^	1.00	0.72	0.71
Professional	0.71	.87	N/A	N/A	1.00	0.49
Usability	0.71	.78	N/A	N/A	N/A	1.00

^a^Column 1 presents the interrater reliability scores for each category.

^b^Column 2 presents the interitem consistency for each category.

^c^Columns 3 to 6 present the correlations between categories presented in the top right half of the table only.

^d^N/A: not applicable.

**Table 3:** Item statistics by category for final version of the Mobile App Rubric for Learning.

Category and item		Cronbach α	Values, mean (SD)
**Teaching and learning**		**.91**	**12.82 (6.93)**
	Purpose	.75	2.33 (1.35)
	Pedagogy	.83	1.97 (1.27)
	Generates learning	.90	1.61 (1.05)
	Quantity of information	.85	1.80 (1.39)
	Relevance to study	.88	1.90 (1.04)
	Instructional features	.76	1.15 (1.21)
	User interactivity	.57	1.29 (0.95)
	Feedback	.40	0.76 (0.88)
	Efficiency	.93	1.42 (1.16)
**User centered**		**.96**	**10.89 (7.17)**
	Subjective quality	.93	1.24 (1.13)
	Satisfaction	.95	1.51 (1.23)
	Perceived usefulness	.92	1.65 (1.15)
	Perceived importance	.90	1.47 (1.03)
	User Experience	.81	2.18 (1.00)
	Intention to reuse	.94	1.33 (1.22)
	Engagement	.91	1.52 (1.12)
**Professional**		**.74**	**5.94 (3.40)**
	In line with standards	.79	2.46 (1.02)
	Credibility	.82	2.94 (1.60)
	Information quality	.83	1.43 (1.54)
**Usability**		**.82**	**16.47 (4.49)**
	Aesthetics	.83	2.47 (0.93)
	Functionality	.70	3.01 (0.94)
	Differentiation	.76	1.40 (0.87)
	Ease of use	.70	2.92 (0.80)
	Advertisements	.36	3.73 (0.86)
	Technical specifications	.61	1.23 (0.99)
	Advantage of app	.87	1.71 (1.07)

The final version of the MARuL rubric (version 4) is provided in Multimedia Appendix 2.

## Discussion

### Principal Findings

The rigorous development of the MARuL has provided a robust and reliable instrument that can be used by medical students and their teachers to evaluate the value of apps to support just-in-time medical student learning. Potential rubric items were identified from a literature search; and medical students, the end users, identified relevant items via a structured nominal group technique. The preliminary instrument was refined, directed by analysis of internal consistency and interrater reliability, and the final MARuL instrument showed acceptable reliability and usability. Although rubrics for the evaluation of education and health apps are common, they tend to be generic [11,12]. We have developed a specific instrument for evaluating the value of medical education apps for learning.

We took a multidimensional approach to developing the MARuL in line with guidance from the literature to date [10,12]. Where appropriate, we adapted items, with acknowledgment, from extant instruments like the MARS. The MARS is a literature-informed, rigorously developed instrument that is widely used in the evaluation of health apps [10]. Our rubric measures the overall value of an app and represents the overall and category scores. This allows evaluation of how the app performs in different domains of value. To our knowledge, the use of multiple items to assess *professional measures* is unique to this rubric; medicine emphasizes the importance of a credible and reliable source of information when informing student learning or patient education. The other 3 categories—*teaching and learning*, *user centered*, and *usability*—are common among evaluative measures [11,12]. These were included in the rubric because students agreed that they address aspects of apps that contribute to valuable student learning. During testing for the internal consistency of each category of the MARuL, cost was observed to be negatively related to the other items in the usability category. That is, apps with a high cost received a low score for the item, as students were less likely to pay for more expensive apps, but a high cost was related to high scores for other usability items. Cost remains an important consideration when choosing an app for student learning, as a lower cost is desirable, for individual students or institutional purchase, but a balance must be maintained between cost and other aspects of usability. Therefore, cost is captured in the basic information about each app (Multimedia Appendix 2), which is also the approach taken in the MARS [10].

The strength of this design is our focus on the end users, medical students, as the main source of input in the development of this rubric. This contrasts with most measures that rely on experts in education and technology to develop rubrics for evaluating apps [10,19]. Although special expertise is important when developing an accurate and reliable rubric, we believe the user voice is as important, if not more so, to ensure that the rubric is fit for purpose. Using a modified nominal group technique and student participants, we were able to confirm that the current literature surrounding evaluation of apps corresponded to student ideas about what made apps valuable to them in their learning. It also gives the user ownership of the means to evaluate the value of the technology they will be accessing.

Just-in-time learning is a common practice used by medical students in both the clinical environment and study situations [39]. Just-in-time learning especially relates to clinical skills, as medical students are constantly refining their history taking and physical examination skills with peers, in simulated clinical encounters, and with real patients. At an average of 8 min to complete after trialing an app, the MARuL rubric is easy to use and provides a fast evaluation of apps for learning.

### Limitations

Although care was taken in the development and implementation of this project, there were some potential limitations. One researcher (GT) reviewed the final set of articles generated from the literature search to develop the list of potential evaluation criteria. As many articles were included, this was a large task, and it is possible that potential evaluation terms were overlooked and not extracted. Having two researchers consult the literature would have minimized this possibility; however, the number of terms extracted and their overlap, plus the nominal group’s lack of additions during the initial ranking of terms, gives us confidence that the evaluation terms extracted from the literature search was comprehensive.

The recruitment of students to the nominal group was by convenience sampling. Research team members asked students to participate in the group. Every effort was made to recruit a variety of students, but the timing of the nominal group in the summer holidays limited the number of students available to take part. A related potential limitation is that students in the nominal group may not have felt confident in rejecting many of the criteria found during the literature review because of concerns around power and hierarchy. However, this was mitigated by having a medical student researcher facilitate the nominal group with another research team member, with no direct effect on student assessment, acting in a support role.

Finally, there are potential limitations because of the smaller than anticipated number of apps reviewed to complete the reliability testing. However, although eight apps identified in the original search were no longer available at the time of rubric testing, the total number reviewed was still acceptable for reliability analysis.

### Next Steps

Our next steps for this research include further refinement of the rubric and construct validity testing to compare the MARuL with measures that evaluate health-related apps and education-oriented apps to measure convergent and discriminant validity. We have also adapted the language to create a student version of the rubric and plan to use it to look at the relationship between student evaluation of app value and teacher evaluation of app value. Finally, we believe that with minor changes to the language of some of the items, the rubric can be used with other types of health professional learning apps, for example, apps that focus on student learning of orthopedic skills.

### Conclusions

The MARuL is a quick and user-friendly method that teachers can use to evaluate the value of an app for just-in-time learning. Through the inclusion of both experts and student stakeholders in the development process, it should be a robust method for teachers to use when deciding whether to download an app to recommend to students for just-in-time clinical skills learning.

## Appendix

Multimedia Appendix 1 Terms used to evaluate the value of apps and/or mobile technology.

Multimedia Appendix 2 Final version of the Mobile App Rubric for Learning (MARuL).

## Abbreviations

e-learning: electronic learning
ICC: intraclass correlation coefficient
MARS: Mobile App Rating Scale
MARuL: Mobile App Rubric for Learning
