# Supplementary material for: A Tool for Rating the Value of Health Education Mobile Apps to Enhance Student Learning (MARuL): Development and Usability Study
Source: JMIR Mhealth Uhealth. 2020 Jul 31;8(7):e18015. doi: 10.2196/18015 (PMC7428912; doi:10.2196/18015)
Supplement: Multimedia Appendix 1 [file mhealth_v8i7e18015_app1.pdf]

* denotes sub term only mentioned in article

Main term	Subterms	Literature
Ease of use	Navigation within app<br>User friendliness<br>Perceived ease of use<br>Intuitiveness	Jonas-Dwyer 2012; Hurst 2018; Paglialonga 2018; Böhme 2017; Fiore 2017; Cherner 2016; Arzola 2016; Lee & Cherner 2015; Franko 2015; Lee & Kim 2015; Rosell-Aguilar 2017*; Perry 2016*; Israelson 2015*; McArthur 2018; Ok 2015*; Pilcher 2016; Stoyanov 2015; Davies 2012*; Joo 2011*; Green 2014*; Kalz 2014; Mustaffa 2000; Simões 2018
Perceived usefulness	Perceived quality of content and function<br>Need	Davies 2012; Joo 2011; Paglialonga 2018*; Hurst 2018*;
Perceived importance		Davis 1989
Perceived enjoyment		Wang 2018
Functionality	System quality<br>Media integration<br>Stability<br>Reliability<br>Performance<br>Consistent (across learners, time, devices)<br>Ability to save progress<br>Usability<br>Connectivity (usable without internet)<br>Modification of data put into application<br>Customisable (personalisable)<br>Export features<br>Disability features (accessible features)<br>Guidance<br>Adjustability<br>Gestural design	Jonas-Dwyer 2012; Hurst 2018*; Paglialonga 2018; Böhme 2017; Fiore 2017; Cherner 2016; Arzola 2016; Soad 2016; Torous 2016*; Baloh 2015; Lee & Cherner 2015*; Franko 2015; Stoyanov 2015; Aungst 2014*; DiFilippo 2018; Franko 2012; Green 2014*; Kalz 2014*; Martín-Monje 2014*; Wang 2018; Papadakis 2017; Jin 2015*; Lee & Kim 2015*; Traxler 2007*; Ok 2015*; Pilcher 2016; Chen 2016*; Vincent 2012; Perry 2016*; Baran 2017*; Velsen 2013*; Penzenstadler 2016*; Rosell-Aguilar 2017*; Singh 2016*; Mustaffa 2000; Simões 2018
Aesthetics	Design<br>Screen design<br>Information presentation<br>User interface	Jonas-Dwyer 2012*; Hurst 2018*; Paglialonga 2018; Böhme 2017; Fiore 2017*; Cherner 2016; Arzola 2016;

	Included features<br>Graphics<br>Layout<br>Visual appeal	Jin 2015*; Lee & Cherner 2015; Franko 2015; Lee & Kim 2015; Rosell-Aguilar 2017*; Perry 2016*; Israelson 2015; Pilcher 2016; McArthur 2018*; Jin 2015; Mustaffa 2000; Franko 2012; Papadakis 2017*; Martín-Monje 2014*; Kalz 2014*; Stoyanov 2015; Baran 2017; Simões 2018; Vincent 2012*
Efficiency	Economical efficiency<br>Proportionate, that is, not more ponderous, onerous, or time-consuming than the learning experience or the delivery and implementation of the learning itself<br>Productivity	Cherner 2016; Lee & Kim 2015*; Traxler 2007; Soad 2016;
Credibility of developers	Involvement of health professionals in developing the app<br>Authority<br>Affiliation	Jonas-Dwyer 2012; Hurst 2018*; Paglialonga 2018*; Böhme 2017; Fiore 2017; Arzola 2016*; Franko 2015*; Soad 2016; Butcher 2015; Penzenstadler 2016*; Singh 2016; Aungst 2014*; Jin 2015*; Simões 2018*; Velsen 2013*; Pilcher 2016; Ok 2015;
User ratings	User reviews<br>Recommendation from other users<br>Worth recommending	Hurst 2018*; Böhme 2017; Simões 2018; Singh 2016; McArthur 2018; Stoyanov 2015
Engagement	Stimulate interest in topic	Böhme 2017; Cherner 2016; Arzola 2016; Lee & Cherner 2015; Lee & Kim 2015; Rosell-Aguilar 2017; Israelson 2015; McArthur 2018; Paglialonga 2018; Stoyanov 2015; Vincent 2012; Singh 2016; Simões 2018; Liu 2017; Pilcher 2016; Papadakis 2017; Ok 2015; Baran 2017; Mustaffa 2000;
Information quality	Accuracy<br>Evidence base	Hurst 2018*; Paglialonga 2018; Böhme 2017; Fiore

	Credibility<br>Source<br>Contextuality	2017*; Cherner 2016*; Arzola 2016; Torous 2016*; Jin 2015*; Israelson 2015*; Ok 2015*; Stoyanov 2015; Wang 2018; Aungst 2014*; DiFilippo 2018; Green 2014*; Kalz 2014; Martín-Monje 2014; Baran 2017*; Chen 2016; Butcher 2015*; Pilcher 2016*; Penzenstadler 2016; Liu 2017; Perry 2016; Velsen 2013; Vincent 2012*; Rosell-Aguilar 2017; Franko 2015; Mustaffa 2000; Simões 2018
Subjective quality		Böhme 2017; Arzola 2016; Perry 2016; Paglialonga 2018; Stoyanov 2015;
Satisfaction		Hurst 2018; Stoyanov 2015; Joo 2011; Wang 2018
Cost	Perceived fee	Jonas-Dywer 2012; Hurst 2018; Fiore 2017; Arzola 2016; Lee & Cherner 2015; Franko 2015; Lee & Kim 2015; Rosell-Aguilar 2017; Perry 2016; McArthur 2018; Payne 2012; Penzenstadler 2018; Singh 2016; Wang 2018*; Vincent 2012; Ok 2015; Stoyanov 2015; Mustaffa 2000
Relevance to study/course	Situatedness and contextualisation<br>Currency<br>Scope of information<br>Actionable/practical<br>Connections to future learning<br>Understandability<br>Authenticity<br>Knowledge support	Jonas-Dwyer 2012*; Hurst 2018*; Fiore 2017; Cherner 2016; Arzola 2016; Torous 2016*; Jin 2015*; Lee & Cherner 2015; Lee & Kim 2015; Rosell-Aguilar 2017; McArthur 2018; DiFilippo 2018*; Green 2014; Baran 2017; Vincent 2012; Penzenstadler 2016*; Stoyanov 2015*; Perry 2016*; Böhme 2017*; Pilcher 2016; Papadakis 2017; Traxler 2007; Mustaffa 2000

Quantity of information	Comprehensiveness	Arzola 2016; Perry 2016*; Stoyanov 2015; Velsen 2013; Penzenstadler 2016*; Butcher 2015; Baran 2017*; Simões 2018
Instructional features		Lee & Cherner 2015; Cherner 2016; McArthur 2018; Mustaffa 2000; Papadakis 2017; Rosell-Aguilar 2017; Vincent 2012
Feedback	Assessment feature<br>Critical evaluation	Cherner 2016; Arzola 2016*; Lee & Cherner 2015; McArthur 2018; Ok 2015; Green 2014; Baloh 2015; Soad 2016; Chen 2016; Vincent 2012; Baran 2017*; Pilcher 2016; Papadakis 2017; Rosell-Aguilar 2017; Mustaffa 2000
Sharing	Collaboration<br>Communication<br>Community	Cherner 2016*; Arzola 2016; Baloh 2015*; McArthur 2018; Pilcher 2016; Green 2014; Chen 2016; Vincent 2012; Singh 2016; Soad 2016*; Stoyanov 2015; Lee & Cherner 2015; Penzenstadler 2016*; Papadakis 2017; Rosell-Aguilar 2017; Baran 2017; Mustaffa 2000
Advantage of using the app over web-based or conventional equivalent		Jonas-Dwyer 2012; Fiore 2017; Kalz 2014
Privacy of information	Security<br>Safety mechanism<br>Trustworthiness – in terms of information, data protection<br>Confidentiality<br>Benefit vs risk of using app	Jonas-Dwyer 2012; Hurst 2018; Paglialonga 2018; Böhme 2017*; Fiore 2017; Cherner 2016*; Arzola 2016; Torous 2016*; Jin 2015*; Baloh 2015*; Franko 2015; Aungst 2014; Rosell-Aguilar 2017*; Stoyanov 2015*; Soad 2016*; Lee & Cherner 2015*; Singh 2016;
Capacity to generate learning	Skill development potential<br>Learning effectiveness<br>Applicability to individual learning style	Martín-Monje 2014; DiFilippo 2018*; Wang 2018*; Baran 2017*; Traxler 2007*; Lee & Kim 2015*;

	Cognitive development	Lee & Cherner 2015*; Ok 2015*; Mustaffa 2000*
Pedagogy	Knowledge at the right time<br>Cognitive effort<br>Content complexity, integration and management<br>Thinking skills required<br>Promote behaviour change	Soad 2016; Baloh 2015; Rosell-Aguilar 2017; Vincent 2012*; DiFilippo 2018*; Baran 2017; Chen 2016; Mustaffa 2000; Simões 2018*; Lee & Cherner 2015*
Motivation		Cherner 2016; Lee & Cherner 2015; Lee & Kim 2015; McArthur 2018; Ok 2015; Pilcher 2016; Baran 2017; Chen 2016; Papadakis 2017; Rosell-Aguilar 2017; Martín-Monje 2014; Liu 2017; Mustaffa 2000
Self-directedness		Lee & Kim 2015
Intention to reuse	Frequency of use	Cherner 2016*; McArthur 2018; Stoyanov 2015; Wang 2018
User experience	Entertainment	Hurst 2018; Fiore 2017; Stoyanov 2015*; McArthur 2018*; Rosell-Aguilar 2017; Paglialonga 2018; Simões 2018*
Purpose	Intended audience<br>Multipurpose<br>Goals	Böhme 2017; Fiore 2017; Cherner 2016*; Arzola 2016; Ok 2015; Pilcher 2016; Stoyanov 2015*; Rosell-Aguilar 2017; Kalz 2014; DiFilippo 2018; Simões 2018*
Advertisements (lack of)		Fiore 2017; Cherner 2016; Perry 2016; Vincent 2012; Papadakis 2017; Rosell-Aguilar 2017
Differentiation		Cherner 2016; Lee & Cherner 2015; Pilcher 2016; Papadakis 2017; Rosell-Aguilar 2017; Mustaffa 2000
Product description	App classification	Arzola 2016; Stoyanov 2015
Playfulness		Arzola 2016; Pilcher 2016

In line with professional standards	Legal<br>Ethical	Torous 2016; Lee & Kim 2015*; Penzenstadler 2016; Traxler 2007*
Transparent	Disclosure	Fiore 2017; Torous 2016; Butcher 2015; Penzenstadler 2016*; Liu 2017;
User interactivity		Jonas-Dwyer 2012; Böhme 2017; Fiore 2017; Lee & Cherner 2015; Israelson 2015; Stoyanov 2015; Penzenstadler 2016; Rosell-Aguilar 2017; Martín-Monje 2014; Liu 2017; Baran 2017; Mustaffa 2000; Simões 2018
Technical specifications	Platform<br>Platform integration<br>Syncing between devices<br>Regular updates<br>Compatibility<br>Support<br>Portability<br>Data mobility	Hurst 2018; Fiore 2017*; Cherner 2016*; Arzola 2016*; Soad 2016*; Baloh 2015*; Gaglani 2014*; Lee & Kim 2015*; Martín-Monje 2014; Butcher 2015*; Singh 2016*; Jonas-Dwyer 2012*; Franko 2015*; Böhme 2017*; Perry 2016*; Lee & Cherner 2015*; Vincent 2012*; Pilcher 2016*; Penzenstadler 2016*; Rosell-Aguilar 2017; McArthur 2018; Aungst 2014*; Baran 2017*; Mustaffa 2000

Appendix 1 References

1. Jonas-Dwyer DRD, Clark C, Celenza A, Siddiqui ZS. Evaluating Apps for Learning and Teaching. International Journal of Emerging Technologies in Learning (iJET). 2012;7(1):54-7.
2. Böhme C, von Osthoff MB, Frey K, Hübner J. Development of a Rating Tool for Mobile Cancer Apps: Information Analysis and Formal and Content-Related Evaluation of Selected Cancer Apps. Journal of Cancer Education. 2017:1-6.
3. Simões P, Silva AG, Amaral J, Queirós A, Rocha NP, Rodrigues M. Features, Behavioral Change Techniques, and Quality of the Most Popular Mobile Apps to Measure Physical Activity: Systematic Search in App Stores. JMIR mHealth and uHealth. 2018.
4. Kalz M, Lenssen N, Felzen M, Rossaint R, Tabuenca B, Specht M, et al. Smartphone apps for cardiopulmonary resuscitation training and real incident support: A mixed-methods evaluation study. Journal of Medical Internet Research. 2014;16(3).
5. Franko OI, Andrawis JP, Mickelson DT. Mobile apps for orthopedic surgeons: How useful are they? Orthop Res Rev. 2015;7:39-45.
6. Lee J-S, Kim S-W. Validation of a Tool Evaluating Educational Apps for Smart Education. Journal of Educational Computing Research. 2015;52(3):435-50.

7. Hurst EJ. Evaluating Health and Wellness Mobile Applications. Journal of Hospital Librarianship. 2018.
8. Cherner T, Fegely A, Lee CY, Santaniello L. A detailed rubric for assessing the quality of teacher resource apps. Journal of Information Technology Education: Innovations in Practice. 2016;15(1):117-43.
9. Arzola R, Havelka S. Apps in Higher Education: Criteria and Evaluation. The Charleston Advisor. 2016;17(3):55-7.
10. Fiore P, Kuo A, Borycki E, Lau F, Bliss G, Courtney K, et al. How to evaluate mobile health applications: A scoping review. Studies in Health Technology and Informatics. 2017;234:109-14.
11. Lee C-Y, Cherner TS. A Comprehensive Evaluation Rubric for Assessing Instructional Apps. Journal of Information Technology Education: Research. 2015;14:21-53.
12. McArthur CL, Lubniewski KL. Evaluating Instructional Apps Using the App Checklist for Educators (ACE). lnternational Electronic Journal of Elementary Education. 2018;10(3):323-9.
13. Paglialonga A, Lugo A, Santoro E. An overview on the emerging area of identification, characterization, and assessment of health apps. Journal of Biomedical Informatics. 2018;83:97-102.
14. Pilcher J. Mobile Apps for Educational Purposes. Journal for Nurses in Professional Development. 2016;32(6):306-8.
15. Stoyanov SR, Hides L, Kavanagh DJ, Zelenko O, Tjondronegoro D, Mani M. Mobile App Rating Scale: A New Tool for Assessing the Quality of Health Mobile Apps. JMIR mHealth and uHealth. 2015;3(1):e27.
16. Mustaffa FY, Salam AR, Muhammad F, Bunari G, Asary LH. Literature Review of Educational App Evaluation Rubrics. 11th Language for Specific Purposes International Conference & 10th Global Advances in Business Communication Conference. 2000.
17. Ok MW, Kim MK, Kang EY, Bryant BR. How to Find Good Apps. Intervention in School and Clinic. 2015;51(4):244-52.
18. Perry R, Lunde B, Chen KT. An evaluation of contraception mobile applications for providers of family planning services. Contraception. 2016;93(6):539-44.
19. Rosell-Aguilar F. State of the app: A taxonomy and framework for evaluating language learning mobile applications. CALICO Journal. 2017;34(2):243-58.
20. Green LS, Hechter RP, Tysinger PD, Chassereau KD. Mobile app selection for 5th through 12th grade science: The development of the MASS rubric. Computers & Education. 2014;75:65-71.
21. Davies BS, Rafique J, Vincent TR, Fairclough J, Packer MH, Vincent R, et al. Mobile Medical Education (MoMEd) - how mobile information resources contribute to learning for undergraduate clinical students - a mixed methods study. BMC Medical Education. 2012.
22. Joo YJ, Lim KY, Kim EK. Online university students' satisfaction and persistence: Examining perceived level of presence, usefulness and ease of use as predictors in a structural model. Computers & Education. 2011.
23. Israelson MH. The App Map. Read Teach. 2015;69(3):339-49.
24. Davis FD. Perceived Usefulness, Perceived Ease of Use, and User Acceptance of Information Technology. MIS Quarterly. 1989.
25. Wang Y-Y, Wang Y-S, Lin H-H, Tsai T-H. Developing and validating a model for assessing paid mobile learning app success. Interactive Learning Environments. 2018;3(6):1-20.
26. Soad GW, Duarte Filho NF, Barbosa EF. Quality evaluation of mobile learning applications. 46th Annual Frontiers in Education Conference, FIE 2016. 2016;2016.

27. Baloh M, Zupanc K, Kosir D, Bosnić Z, Scepanović S, Jurisic D, et al. A quality evaluation framework for mobile learning applications. 4th Mediterranean Conference on Embedded Computing, MECO 2015. 2015:280-3.
28. Papadakis S, Kalogiannakis M, Zaranis N. Designing and creating an educational app rubric for preschool teachers. Educ Inf Technol. 2017;22(6):3147-65.
29. Vincent T. Ways to Evaluate Educational Apps. Learning in Hand with Tony Vincent. 2012;2018.
30. DiFilippo KN, Huang W-HD, Chapman-Novakofski KM. Mobile Apps for the Dietary Approaches to Stop Hypertension (DASH): App Quality Evaluation. Journal of Nutrition Education and Behavior. 2018;50(6):620-5.
31. Franko OI, Tirrell TF. Smartphone App Use Among Medical Providers in ACGME Training Programs. Journal of Medical Systems. 2011.
32. Martín-Monje E, Arús J, Rodríguez Arancón P, Calle-Martínez C. REALL: Rubric for the evaluation of apps in language learning. 2014.
33. Aungst TD, Clauson KA, Misra S, Lewis TL, Husain I. How to identify, assess and utilise mobile medical applications in clinical practice. Int J Clin Pract. 2014;68(2):155-62.
34. Jin M, Kim J. Development and Evaluation of an Evaluation Tool for Healthcare Smartphone Applications. Telemedicine and e-Health. 2015;21(10):831-7.
35. Traxler J. Defining, Discussing, and Evaluating Mobile Learning: The moving finger writes and having writ. 2007.
36. Singh K, Drouin K, Newmark LP, Rozenblum R, Lee J, man A, et al. Developing a Framework for Evaluating the Patient Engagement, Quality, and Safety of Mobile Health Applications. Issue Brief (Commonw Fund). 2016;5:1-11.
37. Chen X. Evaluating Language-learning Mobile Apps for Second-language Learners. Journal of Educational Technology Development and Exchange. 2016;9(2).
38. Baran E, Uygun E, Altan T. Examining Preservice Teachers’ Criteria for Evaluating Educational Mobile Apps. Journal of Educational Computing Research. 2017;54(8):1117-41.
39. Penzenstadler L, Chatton A, Van Singer M, Khazaal Y. Quality of Smartphone Apps Related to Alcohol Use Disorder. European Addiction Research. 2016;22(6):329-38.
40. Torous JB, Chan SR, Yellowlees PM. To use or not' Evaluating ASPECTS of smartphone apps and mobile technology for clinical care in psychiatry. Journal of Clinical Psychiatry. 2016;77(6):e734-e8.
41. Velsen L, Beaujean DJMA, Gemert-Pijnen JEWC. Why mobile health app overload drives us crazy, and how to restore the sanity. BMC Medical Informatics and Decision Making. 2013.
42. Butcher R, MacKinnon M, Gadd K, LeBlanc-Duchin D. Development and Examination of a Rubric for Evaluating Point-of-Care Medical Applications for Mobile Devices. Medical Reference Services Quarterly. 2015;34(1):75-87.
43. Liu AG, Altman BA, Schor K, Strauss-Riggs K, Thomas TN, Sager C, et al. Proposing a Framework for Mobile Applications in Disaster Health Learning. Disaster med public health prep. 2017;11(04):487-95.
44. Payne KBF, Wharrad H, Watts K. Smartphone and medical related App use among medical students and junior doctors in the United Kingdom (UK): a regional survey. BMC Medical Informatics and Decision Making. 2012.
45. Stoyanov SR, Hides L, Kavanagh DJ, Zelenko O, Tjondronegoro D, Mani M. Mobile App Rating Scale: A New Tool for Assessing the Quality of Health Mobile Apps. JMIR mHealth and uHealth. 2015.
46. Gaglani SM, Topol EJ. iMedEd: The Role of Mobile Health Technologies in Medical Education. Academic medicine : journal of the Association of American Medical Colleges. 2014.
