# Supplementary material for: A Tool for Rating the Value of Health Education Mobile Apps to Enhance Student Learning (MARuL): Development and Usability Study
Source: JMIR Mhealth Uhealth. 2020 Jul 31;8(7):e18015. doi: 10.2196/18015 (PMC7428912; doi:10.2196/18015)
Supplement: Multimedia Appendix 2 [file mhealth_v8i7e18015_app2.pdf]

Mobile app rubric for learning (MARuL)

This rubric is made up of items in four categories: user engagement, teaching and learning, usability, and app professionalism. These are thought to encompass value for just-in-time learning as determined by students.

For each of the following items, think about the clinical skills learning your students need and rate the app based on the guiding questions and their related standards. The items are rated on a 5-point Likert scale (0=does not fulfil the item requirements, 1=poorly fulfils requirements, 2=somewhat fulfils requirements, 3=mostly fulfils requirements, 4=fully meets requirements). **Choose the standard that most closely matches your feeling about the app in relation to that item even if it does not quite fit the full description.**

At the end of each section add up the scores you gave each item to get the total score for the section.

Basic Information

For the following items, please check the app information on the appropriate app store.

Which mobile device is this app on? Android / iOS

Please type the name of the App ________________________________

Please type the name of the App Developer ________________________________

What is the app version number? ________________________________

When was it last updated? ________________________________

How much does the app cost? ____________________________ (monthly / yearly / one-off)

Does the app work on your device? Yes No

Part A: Teaching & Learning Measures

These measures relate to aspects of teaching and learning such as pedagogy, relevance to study, interactivity and efficiency.

Purpose: Consider the following kinds of questions as you make your decision: What is the purpose of the app? Does it align with the learning the student requires? Who is the intended audience of the app?

0. App purpose is not related to clinical skills learning. Intended audience is not medical students.
1. App purpose may be related to clinical skills learning but does not provide the learning required. Intended audience may be other students such as nursing or dental students, or teachers.
2. App purpose is related to clinical skills. Aimed at medical students but does not provide all of the learning that is needed.
3. App purpose is related to clinical skills and provides the learning and skills that are required.

4. App's purpose is to teach clinical skills. App has been made specifically for medical students.

Pedagogy (adapted from Chen, 2016): Consider the following kinds of questions as you make your decision: Is the learning provided by the app consistent with the learning goals I have for my students? Does the app support 'just-in-time' learning?

0. Content of the app is not consistent with the learning goals I have for my students. It's not targeted towards just-in-time learning
1. Content of the app is not consistent with learning goals I have for my students but could be targeted to just-in-time learning.
2. Content of the app target goals related to those I have for my students. App could not be used for just-in-time learning
3. Content of the app target goals related to those I have for my students. App could be used for just-in-time learning
4. Content of the app are consistent with learning goals I have for my students, and the app can definitely be used for just-in-time learning.

Capacity to generate learning (adapted from Martín-Monje, 2014): Consider the following kinds of questions as you make your decision: Does the app support achievement of learning goals I have for my students? Does it feel as if the app could teach them something?

0. Does not support the achievement of learning goals. Students couldn't learn anything from using the app.
1. Helps learning but may not directly achieve learning goals
2. Supports achievement of learning goals
3. Generates worthwhile learning and achieves learning goals
4. Fully supports achievement of learning goals and students could definitely learn from using the app.

Quantity of information (Adapted from Stoyanov, 2015): Consider the following kinds of questions as you make your decision: Is the quantity of information adequate for its intended use? Is it at the appropriate level of detail for the learner yet comprehensive for its intended use?

0. Minimal or overwhelming amounts of information with poor level of detail
1. Insufficient or possibly overwhelming amount of information with inappropriate level of detail
2. Amount of information is appropriate, but does not have appropriate level of depth
3. Broad range of information. May have gaps or unnecessary detail
4. Information is both comprehensive and concise.

Relevance to study/course: Consider the following kinds of questions as you make your decision: Is the information on this app relevant to my students' study needs? Does it align with the course material?

0. App does not appear to have relevant content
1. Some content on the app may be relevant to study
2. Most content on the app is relevant to study, but may not be comprehensive enough
3. App content is relevant to study and course, may still need other study aids.
4. App content is highly relevant to study and could be used as a sole study resource

Instructional features: Consider the following kinds of questions as you make your decision: Does the app include features such as instructions, tutorials or videos?

0. App has no instructional features
1. App attempts to have instructional features. These may not be appropriate to the content
2. App has functional instructional features
3. App's instructional features are useful and appropriate
4. App has comprehensive instructional features appropriate to the type of content

User interactivity (Adapted from Stoyanov, 2015): Consider the following kinds of questions as you make your decision: Does the app allow user input, give feedback, prompts, and are these customisable?

0. There are no interactive features of the app and/or no response to interaction
1. Insufficient interactivity or user input options. Lack of feedback
2. App has basic interactive features
3. App has a variety of interactive features
4. App has high level of interactivity through its interactive features, feedback and user input options

Feedback (adapted from Lee et al, 2015): Consider the following kinds of questions as you make your decision: Does the app ask questions, and provide responses on accuracy of an answer? This can range from simple to very detailed information.

0. App does not ask questions, or asks questions but does not provide any feedback
1. App provides minimal feedback, such as incorrect/correct answer with no explanation
2. App provides feedback with an attempt at constructive explanations.
3. App provides feedback that explains answers properly and to an appropriate level
4. App provides feedback with detailed explanations and may provide links or recommendations to resources with more explanation.

Efficiency: Consider the following kinds of questions as you make your decision: Would the app increase the effectiveness of learning for my students? Would it make it easier and quicker for my students to learn?

0. App is unlikely to make student learning more effective or make it easier or quicker for students to learn.
1. App could increase the effectiveness of learning in some ways but without significant impact.
2. App is somewhat likely to increase the effectiveness and ease of learning.
3. App is likely to increase effectiveness and ease of learning, it is a worthwhile resource.
4. App is highly likely to increase effectiveness and ease of learning and is overall a very efficient way to learn the skills needed.

Teaching and Learning total score = __________/36

Part B: User-Centred Measures

User centred measures relate to the way the app engages the user, in terms of perceived usefulness, importance, satisfaction, user experience, subjective quality and intention to reuse.

Subjective quality (Adapted from Stoyanov, 2015): Consider the following kinds of questions as you make your decision: Does the app seem like a good app? Does it have good

ratings? Does it look like something my students might use? Would I recommend the app to students?

0. Would not recommend this app to anybody. Would not use the app at all.
1. Might recommend app to few people, might use app very infrequently.
2. There are several people the app could be recommended to. Might use app infrequently.
3. App worth recommending to most people. Would use regularly.
4. Would recommend the app to most people. I would use the app very regularly.

Satisfaction: Consider the following kinds of questions as you make your decision: Am I happy with what the app does? Would it help motivate my students and help them achieve their goals in my module?

0. This app does not satisfy my expectations and would not be useful to students.
1. The app is somewhat satisfying
2. The app is moderately satisfying
3. The app is mostly satisfying
4. The app satisfies all my expectations and would be very useful to students

Perceived usefulness: Consider the following kinds of questions as you make your decision: Does the app seem like it would be useful to my students and help them with their learning?

0. App does not look like it will enhance or supplement student learning
1. App looks like it has little potential to enhance / supplement student learning
2. App has some potential to enhance / supplement student learning
3. App shows good potential to enhance and supplement student learning
4. App shows strong potential to enhance / supplement student learning

Perceived importance: Consider the following kinds of questions as you make your decision: Does the app appear to be an important part of student learning? Does it look like a resource that would improve my student's study routine?

0. App does not appear to be important for learning and study.
1. App shows minimal potential to be important for learning and study
2. App may have some importance to learning and study
3. App largely fits purpose (theoretically/on paper) and could become part of routine
4. The app appears important and is likely to become part of a study routine

User experience: Consider the following kinds of questions as you make your decision: Does the app perform the way I expect it to?

0. The app does not perform at all in the way I expect it to.
1. The app's performance is poor compared to my expectations.
2. The app has acceptable performance compared to my expectations.
3. The app performs exactly as I expect it to.
4. The app's performance exceeds my expectations.

Intention to reuse: Consider the following kinds of questions as you make your decision: After trying the app do I think a student would use the app again?

0. A student would not use the app again
1. A student would consider using the app again
2. A student would use the app infrequently
3. A student would use the app occasionally

4. A student would use the app frequently and make it part of their regular study

Engagement (Adapted from Stoyanov, 2015): Consider the following kinds of questions as you make your decision: Is the app interesting, fun, and stimulating?

0. The app is not at all engaging
1. The app is slightly engaging
2. The app is somewhat engaging
3. The app is engaging
4. The app is completely engaging

User-centred total score = __________/28

Part C: Professional Measures

Specific to the medical aspect of apps, professional measures include features of apps such as information quality, transparency and credibility of developers.

In line with professional standards: Consider the following kinds of questions as you make your decision: Is the app content consistent with current standards? Is the content up to date?

0. Content does not appear to be in line with current standards
1. A small amount of content appears up to date with current standards
2. Content appears somewhat up to date with current standards
3. Most of the content appears up-to-date with current standards
4. All content appears up-to-date with current standards.

Credibility of app: Consider the following kinds of questions as you make your decision: Were there medical professionals involved in the development of the app? If not, do the app developers have any qualifications or experience in the subject matter of the app?

0. No information is available.
1. App developers have not involved medical professionals.
2. App developers have involved professionals with loosely relevant qualifications.
3. App developers have involved medical professionals.
4. App developers are qualified medical professionals.

Information quality (Adapted from Stoyanov, 2015): Consider the following kinds of questions as you make your decision: Does the app reference information, include credible sources, and incorporate evidence based, accurate content?

0. The source of information is not stated on the app or in the description
1. Source of information is not readily available. The accuracy of the information is unknown
2. The source of the information is available, but accuracy is unclear
3. Source of information is available and seems accurate
4. Source of information on app is easily available and is credible. Information is correct and accurate.

Professional total score = __________/12

Part D: Usability Measures

Usability measures encompass the way the app works, how it looks and other features such as ease of use and cost.

Aesthetics (Adapted from Stoyanov, 2015): Consider the following kinds of questions as you make your decision: Does the design of the app, its interface, information presentation, graphics, and layout help or hinder its use?

0. Design and layout make the app almost unusable
1. Design and layout are poor and make it difficult to use the app. Graphics hinder understanding
2. Design and layout are functional but would not engage the student
3. Design is clear and user is able to navigate layout and information easily.
4. App interface is professional with quality graphics and organization of information. Layout is intuitive.

Functionality (Adapted from Stoyanov, 2015): Consider the following kinds of questions as you make your decision: How well does the app work in terms of accuracy, ease of performing tasks, speed, and performance?

0. App does not work well. May crash or be extremely slow.
1. Some functions of the app work. Still contains major technical problems
2. App works overall. Some functions may be slow and performance may be poor at times.
3. App is mostly functional with minor problems. Speed is not a noticeable issue.
4. App works very well, has timely responses and no technical bugs.

Differentiation: Consider the following kinds of questions as you make your decision: Can different levels of difficulty be set or specific skills to study targeted?

0. There are no options for setting difficulty levels or targeting skills
1. There are limited options for setting difficulty or targeting skills, but not both
2. There are either options for setting difficulty level or targeting skills, but not both
3. There are limited options for both setting difficulty level and for targeting skills
4. There are a wide range of options for both levels of difficulty and targeted skills learning

Ease of use (Adapted from Stoyanov, 2015): Consider the following kinds of questions as you make your decision: How easy is it to learn how to use the app? How clear are the icons/menus and instructions?

0. Learning how to use app is complicated. Icons and instructions are confusing
1. Useable after a lot of time and effort. Navigation is poor.
2. Useable after some time/effort. Navigation is not particularly intuitive.
3. Easy to use. Somewhat intuitive and user friendly.
4. Perfectly logical, easy, clear and intuitive screen flow throughout app

Advertisements (lack of): Consider the following kinds of questions as you make your decision: Are there advertisements on the app? Do they inhibit easy use of the app? Does paying for a full version or subscription remove the ads?

0. App has ads on every screen which inhibit easy use of the app. Paying for the app to remove ads is not possible
1. App has ads on every screen which inhibit easy use of the app. Paying for app removes ads.

2. App’s ads are not intrusive and do not inhibit easy use of app. Paying for the app to remove ads is not possible
3. App’s ads are not intrusive and do not inhibit easy use of app. Paying for app removes ads.
4. App does not have ads.

Technical specifications (Adapted from Stoyanov, 2015): Consider the following kinds of questions as you make your decision: Is support available? Is the app updated regularly?

0. App does not appear to have any support.
1. App has limited support. Updated infrequently.
2. App has been recently updated.
3. Support is available and app is updated regularly.
4. App support is comprehensive. Updates are regular and address any issues.

Advantage of using app over web-based or conventional equivalent: Consider the following kinds of questions as you make your decision: Is there an advantage to using the app over a resource on the internet or a textbook? For example, interactivity, portability?

0. There is no advantage to using the app
1. There is a minor advantage to using the app in terms of portability
2. There is some advantage to using the app
3. There is an advantage to using the app.
4. Using the app is clearly better than using other resources

Usability total score = __________/28

Notes.

Final Scores

Part A: Teaching and Learning Score: __________

Part B: User-Centred Score: + __________

Part C: Professional Score: + __________

Part D: Usability Score: + __________ =

Overall Usefulness for Learning Score: __________/104

Rubric References

Chen X. Evaluating Language-learning Mobile Apps for Second-language Learners. Journal of Educational Technology Development and Exchange. 2016;9(2).

Lee C-Y, Cherner TS. A Comprehensive Evaluation Rubric for Assessing Instructional Apps. Journal of Information Technology Education: Research. 2015;14:21-53.

Martín-Monje E, Arús J, Rodríguez Arancón P, Calle-Martínez C. REALL: Rubric for the evaluation of apps in language learning. 2014.

Stoyanov SR, Hides L, Kavanagh DJ, Zelenko O, Tjondronegoro D, Mani M. Mobile App Rating Scale: A New Tool for Assessing the Quality of Health Mobile Apps. JMIR mHealth and uHealth. 2015;3(1):e27.
